# Calcium deposition in osteoarthritic meniscus and meniscal cell culture

**DOI:** 10.1186/ar2968

**Published:** 2010-03-30

**Authors:** Yubo Sun, David R Mauerhan, Patrick R Honeycutt, Jeffrey S Kneisl, H James Norton, Natalia Zinchenko, Edward N Hanley, Helen E Gruber

**Affiliations:** 1 Department of Orthopaedic Surgery, Carolinas Medical Center, PO Box 32861, Charlotte, NC 28232, USA; 2 Department of Biostatistics, Carolinas Medical Center, PO Box 32861, Charlotte, NC 28232, USA

## Abstract

**Introduction:**

Calcium crystals exist in the knee joint fluid of up to 65% of osteoarthritis (OA) patients and the presence of these calcium crystals correlates with the radiographic evidence of hyaline cartilaginous degeneration. This study sought to examine calcium deposition in OA meniscus and to investigate OA meniscal cell-mediated calcium deposition. The hypothesis was that OA meniscal cells may play a role in pathological meniscal calcification.

**Methods:**

Studies were approved by our human subjects Institutional Review Board. Menisci were collected during joint replacement surgeries for OA patients and during limb amputation surgeries for osteosarcoma patients. Calcium deposits in menisci were examined by alizarin red staining. Expression of genes involved in biomineralization in OA meniscal cells was examined by microarray and real-time RT-PCR. Cell-mediated calcium deposition in monolayer culture of meniscal cells was examined using an ATP-induced ^45^calcium deposition assay.

**Results:**

Calcium depositions were detected in OA menisci but not in normal menisci. The expression of several genes involved in biomineralization including ENPP1 and ANKH was upregulated in OA meniscal cells. Consistently, ATP-induced calcium deposition in the monolayer culture of OA meniscal cells was much higher than that in the monolayer culture of control meniscal cells.

**Conclusions:**

Calcium deposition is common in OA menisci. OA meniscal cells calcify more readily than normal meniscal cells. Pathological meniscal calcification, which may alter the biomechanical properties of the knee meniscus, is potentially an important contributory factor to OA.

## Introduction

Osteoarthritis (OA) is a disease characterized by the breakdown of hyaline articular cartilage and the formation of osteophytes. A gradual realization, however, is that OA is not merely a cartilage disease, but a disease of the whole joint [1,2]. The OA synovial membrane and subchondral bone have drawn considerable attention recently. Aberrant gene expression in the OA synovium, OA fibroblast-like synoviocytes and OA subchondral bone has been detected [3-5]. The knee menisci are specialized tissues that play a vital role in load transmission, shock absorption and joint stability. Increasing evidence suggests that the knee meniscus may not be a passive bystander in the disease process of OA.

A previous study examined the incidence of horizontal cleavage lesions of the knee menisci in 100 random necropsy specimens and found that the coincidence of horizontal cleavage lesions and OA was frequent [6]. Another study found among persons with radiographic evidence of OA and knee pain or stiffness that the prevalence of meniscal tears was 63%, but the corresponding prevalence among persons without radiographic evidence of OA and knee pain or stiffness was only 23% [7]. Several studies have demonstrated that meniscal degeneration is a general feature of OA knee joints as revealed by magnetic resonance imaging [8-10] and that meniscal degeneration contributes to joint space narrowing [11]. These findings and observations together suggest that pathological changes have occurred in OA menisci.

Calcium crystals are found in the knee joint fluid of up to 65% of OA patients [12-14]. Calcium crystals are also found in hyaline articular cartilage of OA patients [15-17]. There is compelling evidence indicating that these crystals may worsen joint degeneration. Injection of crystals into the knee joint of dogs induced a severe inflammatory response [18]. In cell culture, crystals stimulated mitogenesis [19,20] and the production of matrix metalloproteinases [21,22] and inflammatory cytokines [23,24]. Several proteins, including ectonucleotide pyrophosphatase/phosphodiesterase 1 (ENPP1), progressive ankylosis homolog (ANKH), tissue nonspecific alkaline phosphatase and transglutaminase-2, have been implicated in pathological calcification in OA hyaline articular cartilage [25-28].

Meniscal calcification is common in calcium pyrophosphate dihydrate crystal deposition disease [29-31]. Studies found that 86% of patients with calcium pyrophosphate dihydrate deposition disease had calcified meniscus [29] and that meniscal calcification increased with age and correlated with cartilage lesions both in patients with no history of arthritis and in cadavers [32,33]. Studies investigating calcification in human OA menisci and OA meniscal cell culture, however, are lacking.

In the present study, we examined calcium deposition in OA menisci and investigated the expression of several genes implicated in the biomineralization biological process, including ENPP1, ANKH and matrix Gla protein. We also examined calcium deposition in the monolayer culture of OA meniscal cells and normal meniscal cells. The main purpose of this study was to test the hypothesis that OA meniscal cells may play a role in pathological meniscal calcification.

## Materials and methods

Dulbecco's modified Eagle's medium (DMEM), fetal bovine serum and stock antibiotic/antimycotic mixture were products of Invitrogen (Carlsbad, CA, USA). Calcium phosphocitrate (CaPC) was prepared according to the methods described [34,35]. ^45^Calcium was obtained from Perkin-Elmer (Boston, MA, USA). All other chemicals are purchased from Sigma (St Louis, MO, USA).

### Meniscal specimens

Meniscal specimens were collected, with the approval of the authors' Institutional Review Board, from eight consecutive unselected OA patients who underwent total joint replacement surgery and from three osteosarcoma patients who underwent lower limb amputation surgery at our medical center. Hyaline articular cartilage specimens were also collected. The need for informed consent was waived since these tissues were surgical waste of routine joint replacement surgery and lower limb amputation surgery, and since there was no patient private information being collected.

### Alizarin red staining analysis

Medial menisci were processed to remove fatty and synovial tissues, and were divided from the middle into two portions. The anterior portion was processed to prepare meniscal cells. The posterior portion was processed for alizarin red staining. Briefly, the posterior portion was fixed in 10% formalin, dehydrated in a graded ethanol series and cleared with xylene. A portion 4 mm wide was transversely excised from the middle part of the specimen, embedded in paraffin and sectioned to obtain transverse sections of the specimen. Another portion 15 mm wide was transversely excised from the middle part of the specimen. This portion was divided at the central level horizontally into two pieces. The lower piece was embedded in paraffin and sectioned to obtain longitudinal sections of the specimen.

These transverse sections (three sections from each meniscus) and longitudinal sections (three sections from each meniscus) of OA and normal menisci were stained with alizarin red. Alizarin red staining was graded on a scale of 0 to 4 by two independent observers in a blinded manner, where 0 = no calcium deposition, 1 = limited number of small-sized or medium-sized single calcium deposits at the edges of the meniscus, 2 = limited number of clusters of small-sized and medium-sized calcium deposits at the edges of the meniscus, 3 = clusters of small calcium deposits inside the meniscus and limited number of clusters of small-sized and medium-sized calcium deposits at the edges of the meniscus, and 4 = clusters of small-sized calcium deposits inside the meniscus and widespread clusters of medium-sized and large-sized calcium deposits at the edges of meniscus.

### Cell preparation

Meniscal cells were prepared from the middle part of the anterior portion of the meniscus. Briefly, a piece of the specimen (20 mm wide) was excised from the anterior portion of the meniscus, minced into small pieces (3 mm × 3 mm), and cultured in 100 mm plates at 37°C in medium containing 0.5% antibiotic/antimycotic solution and 10% serum. Every 3 days, the culture medium was changed.

When meniscal cells reached 80% confluence, they were replated. These meniscal cells were fibroblast in appearance, and there were no differences between the OA meniscal cells and the normal meniscal cells in appearance. These cells produced aggrecan and type II collagen when cultured in a three-dimensional matrix [36].

Hyaline articular chondrocytes were prepared as described above.

### Adenosine-5'-triphosphate (ATP)-induced calcium depositionassay

Cell-mediated calcium deposition was investigated using a well-characterized ATP-induced crystal formation/calcium deposition assay. It has been demonstrated that ^45^calcium uptake in the monolayer culture of hyaline articular chondrocytes is proportional to crystal formation [37,38]. Briefly, meniscal cells (passage two) were plated in 24-well plates at 95 to 100% confluence. On the second day, culture media without serum were added and cells were cultured for 24 hours. On the third day, the culture media were replaced with culture media trace-labeled with 1 μCi/ml ^45^calcium. ATP was added immediately at a final concentration of 1 mM. Cells without ATP treatment or with β-glycerophosphate treatment were used as a control. Forty-eight or seventy-two hours later, culture media were removed, and the cells were washed with cold Hank's balanced salt solution five times and treated with 0.1 N NaOH. The radioactivity of the cell lysate was quantified by liquid scintigraphy and normalized against total protein [37,38]. Assays were run in triplicate and the results averaged.

### RNA extraction and microarray analyses

OA meniscal cells and normal control meniscal cells were plated in 100 mm plates at 85% confluence. On the second day, culture medium containing 1% serum was added and the cells were cultured for 24 hours. Culture medium with 1% serum was changed again and cells were cultured for another 24 hours. Total RNA was extracted from these cells using Trizol reagent (Invitrogen) and subjected to microarray analysis as described previously [39,40]. Briefly, double-stranded DNA was synthesized from RNA samples using a SuperScript double-stranded cDNA synthesis kit (Invitrogen). The DNA product was purified using the GeneChip sample cleanup module (Affymetrix, Santa Clara, CA, USA). cRNA was synthesized and biotin-labeled using a BioArray high yield RNA transcript labeling kit (Enzo Life Sciences, Farmingdale, NY, USA). The product was purified using the GeneChip sample cleanup module and subsequently chemically fragmented. The fragmented, biotinylated cRNA was hybridized to a HG-U133_Plus_2 gene chip using Affymetrix Fluidics Station 400 (Affymetrix).

The fluorescent signal was quantified during two scans by an Agilent Gene Array Scanner G2500A (Agilent Technologies, Palo Alto, CA) and GeneChip operating Software (Affymetrix). GeneSifter software (VizX Labs, Seattle, WA, USA) was used for the analysis of differential gene expression and gene ontology. In the present study, we focused on the differential expression of selected genes that are involved in the biomineralization biological process.

### Real-time RT-PCR

Briefly, cDNA was synthesized using TaqMan^® ^Reverse Transcription Reagents (Applied Biosystems, Inc., University Park, IL, USA). Quantification of relative transcript levels for selected genes and the housekeeping gene GAPDH was performed using the ABI7000 Real Time PCR system (Applied Biosystems, Inc.). TaqMan^® ^Gene Expression assays (Applied Biosystems, Inc.) were used, which contain a FAM-MGB probe for fluorescent detection. cDNA samples were amplified with an initial Taq DNA polymerase activation step at 95°C for 10 minutes, followed by 40 cycles of denaturation at 95°C for 15 seconds and annealing at 60°C for 1 minute. The fold change was calculated and the expression level of genes was normalized to the expression level of GAPDH according to the method described [41]. Each real-time RT-PCR experiment was repeated twice in triplicate and the results averaged.

### Statistical analyses

The difference of alizarin red staining grades between the OA group and the control group was analyzed using the Wilcoxon rank-sum test. The results of cell-mediated calcium deposition assay were expressed as the mean ± standard deviation. The difference of the results between two groups was analyzed using Student's two-sample *t *test. Dose-dependent inhibition of meniscal cell-mediated calcium deposition by CaPC, a potent calcification inhibitor, was analyzed using one-way analysis of variance followed by Tukey's test. In all cases, two-tailed *P *< 0.05 was considered significant. Statistical analysis was performed using the SAS^® ^software, version 9.1 (SAS Institute Inc, Cary NC, USA).

## Results

### Calcium deposition in osteoarthritis menisci

**Figure 1:**
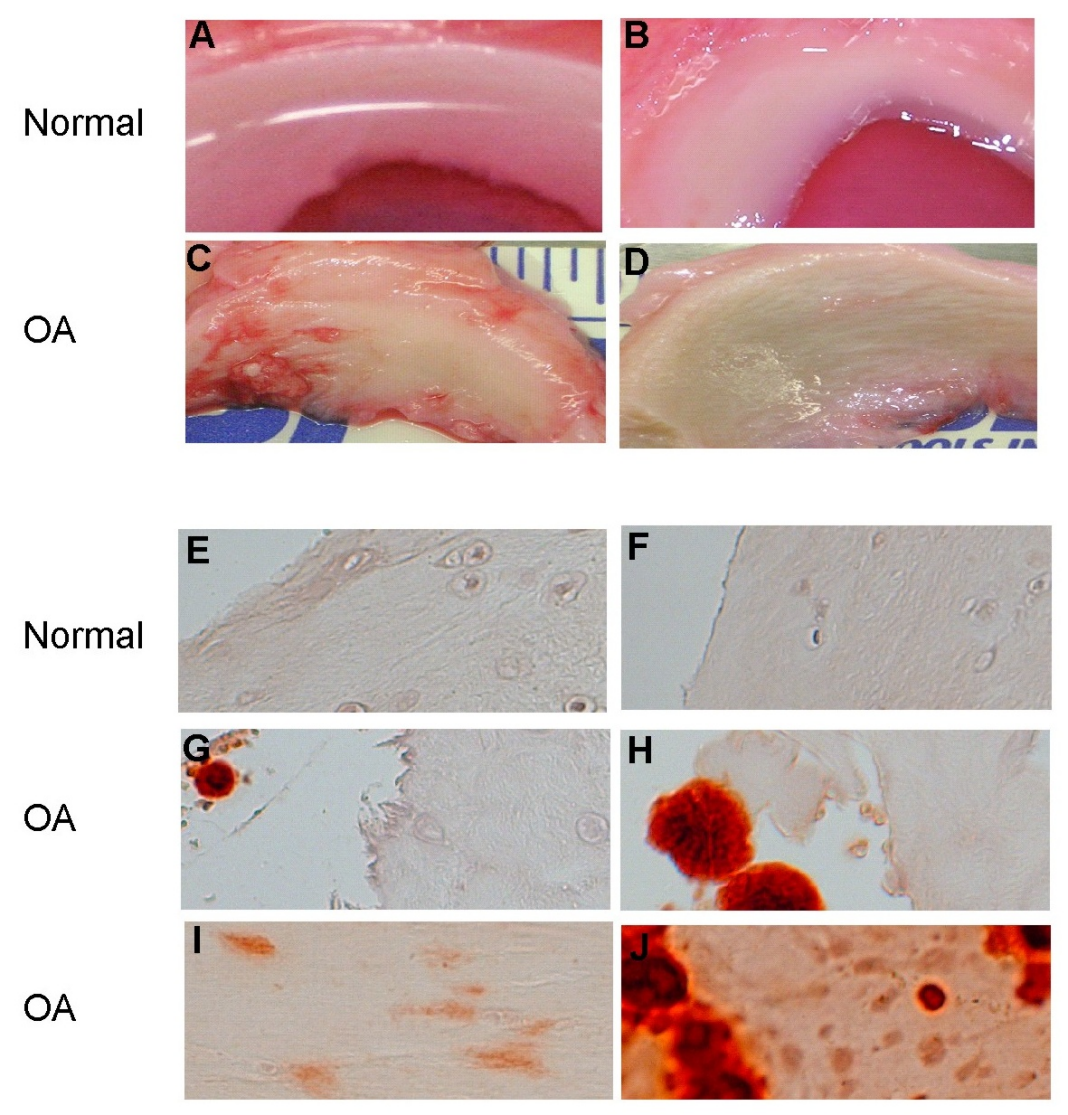
**Normal and osteoarthritis menisci, stained with alizarin red**. Menisci were derived from **(a) **a 39-year-old female osteosarcoma patient, **(b) **a 43-year-old male osteosarcoma patient, **(c) **a 42-year-old male osteoarthritis (OA) patient, and **(d) **a 49-year-old female OA patient. The normal menisci exhibited a smooth, white and glistening surface, with no signs of degeneration (a and b). OA menisci showed discoloration and a rough surface. Degeneration was apparent (c and d). **(e)**, **(f) **There were no calcium depositions in the normal menisci; **(g) **to **(j) **calcium deposition was present in all OA menisci. Representative images of grade 0 (e and f), grade 1 (g), grade 2 (h), grade 3 (i) and grade 4 (j) of alizarin red staining are shown.

Images of two normal control menisci and two age-matched OA menisci are shown in Figure 1. Control menisci have a smooth, white and glistening surface, with no signs of degeneration (Figure 1a, b). In contrast, OA menisci have a rough surface and apparent degeneration (Figure 1c, d). Alizarin red staining demonstrated that calcium deposits existed in all OA menisci derived from eight consecutive unselected OA patients, but not in the normal control menisci (Figure 1e to 1j).

We observed three distinctive patterns of calcium deposition in the OA menisci. The first pattern of calcium deposition was limited numbers of small-sized or medium-sized single calcium deposit (Figure 1g) or small clusters of small-sized and medium-sized calcium deposits (Figure 1h). This type of calcium deposition was almost always found at the edges of the sections of OA menisci and appeared to be associated with meniscal degeneration (Figure 1g, h).

The second pattern of calcium deposition was clusters of small-sized calcium deposits inside the meniscus (Figure 1i). This type of calcium deposition was found in about 65% of the sections among all of the sections of OA menisci.

The third pattern of calcium deposition was widespread clusters of medium-sized and large-sized calcium deposits. This type of calcium deposition was found in about 35% of the sections among all of the sections of OA menisci.

We graded the alizarin red staining according to these patterns as described in Materials and methods. The results along with demographic patient information are presented in Table 1. As shown, calcium deposits existed in the transverse and longitudinal sections of all OA menisci, but not in any sections of the normal control menisci.

**Table 1:** Grade of alizarin red staining

Grade	Normal group			Osteoarthritis group							
		
	12 F	39 F	43 M	42 M	49 F	54 F	55 M	58 F	65 F	66 F	70 F
Transverse section	0	0	0	2	2	3	3	1	3	4	4
Longitudinal section	0	0	0	3	1	3	3	1	4	4	4
Average^a^	0	0	0	2.5	1.5	3	3	1	3.5	4	4

12F, 12-year-old female; 42 M, 42-year-old male; and so forth. The difference of alizarin red staining grades between the osteoarthritis group and the normal control group was statistically significant with *P *< 0.02. ^a^Average of the transverse section grade and the longitudinal section grades.

### Expression of genes implicated in calcification

We examined and compared the expression of ENPP1 and ANKH in OA meniscal cells and in normal meniscal cells. Both microarray and real-time RT-PCR analyses indicated that the expression of ENPP1 and ANKH was upregulated in OA meniscal cells (Table 2). In addition, microarray and real-time RT-PCR analyses indicated that the expression of matrix Gla protein and serglycin, which are putative endogenous calcification inhibitors [42,43], was also upregulated in OA meniscal cells.

**Table 2:** Genes differentially expressed in osteoarthritis meniscal cells compared with normal control cells

Gene name	Gene ID	Real-time RT-PCR^a^	Differential gene expression^b ^(fold)					Description
				
			OA1	OA2	OA3	OA4	OA5	
ENPP1	BF057080	2.1	1.7	2.0	2.8	1.9	2.0	Ectonucleotide pyrophosphatase 1
ANKH	AL833238	1.9	2.4	1.8	1.7	1.5	1.8	Ankylosis, progressive homolog
MGP	NM_000900	5.3	11.8	4.7	21.6	0	17.6	Matrix Gla protein
SRGN	NM_002727	3.3	2.2	3.1	8.3	3.1	1.9	Serglycin

^a^The ratio of the relative expression level of a specific gene in osteoarthritis (OA) meniscal cells derived from five OA patients (RNA mixture) to the relative expression level of the specific gene in the control meniscal cells derived from three normal control subjects (RNA mixture), which were determined by real-time RT-PCR analysis with *P *< 0.01. ^b^Microarray analysis of the differential expression of a specific gene in the individual OA cells derived from five different OA patients compared with three normal control meniscal cells as a group. OA1, derived from a 65-year-old female OA patient; OA2, derived from a 56-year-old female OA patient; OA3, derived from a 50-year-old female OA patient; OA4, derived from a 52-year-old female OA patient; OA5, derived from a 61-year-old male OA patient; Normal 1, derived from a 36-year-old female osteosarcoma patient; Normal 2, derived from a 43-year-old female osteosarcoma patient; Normal 3, derived from a 12-year-old female osteosarcoma patient. Microarray analyses were carried out as described in Materials and methods. The raw microarray data can be found in the Gene Expression Omnibus [GEO:GSE19060] [49].

### Meniscal cell-mediated calcium deposition

**Figure 2:**
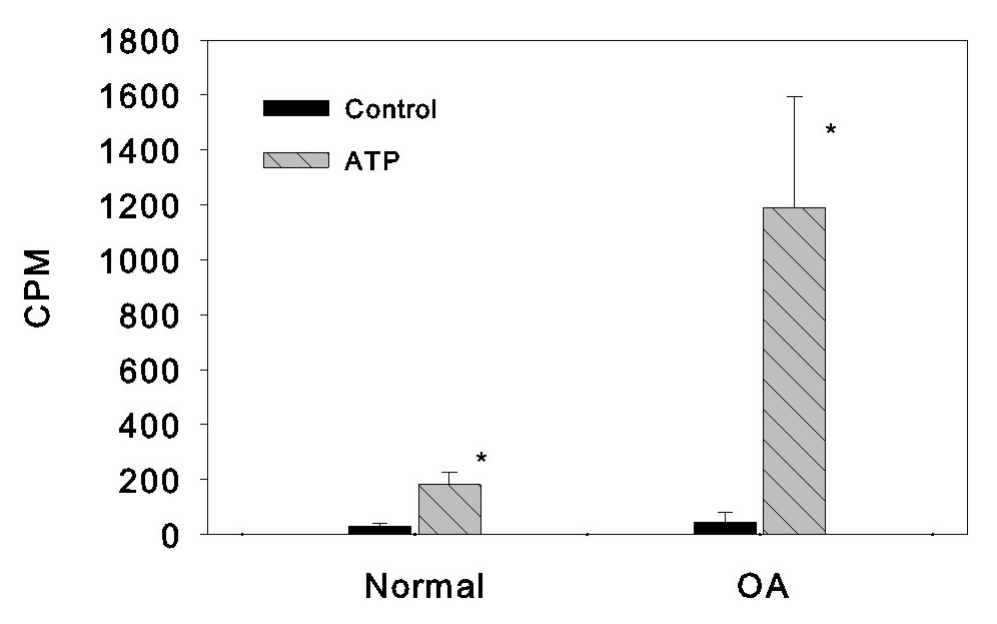
**ATP-induced calcium deposition**. ATP-induced calcium deposition in monolayer cultures of osteoarthritis (OA) meniscal cells derived from five OA patients (right-hand group) was significantly higher than that in the monolayer cultures of normal control meniscal cells derived from three osteosarcoma patients (left-hand group). The difference between the two groups was statistically significant (*P < 0.005). Count per minute (CPM) data were normalized against total protein levels.

Five OA meniscal cell cultures and three normal meniscal cell cultures were investigated using an ATP-induced calcium deposition assay. As shown in Figure 2, ATP induced only a small amount of calcium deposition in the monolayer cultures of normal meniscal cells after treatment with ATP for 48 hours (left-hand group, *P *= 0.006). In contract, ATP induced a large amount of calcium deposition in the monolayer cultures of OA meniscal cells under the same condition (right-hand group, *P *= 0.003). β-Glycerophosphate only induced a small amount of calcium deposition when it was used as an alternative source of phosphate (data not shown). In fact, the ATP-induced calcium deposition in the monolayer cultures of OA meniscal cells derived from five OA patients was more than sixfold greater than that seen in the monolayer cultures of normal meniscal cells derived from three control subjects. The difference between OA meniscal cell-mediated and normal control meniscal cell-mediated calcium deposition was statistically significant (*P *< 0.005). The detailed results of the calcium deposition assay are presented in Table 3.

**Table 3:** Calcium deposition in monolayer cultures of meniscal cells

Normal meniscal cells			Osteoarthritis meniscal cells		
	
Age/gender of patients	Control (CPM)	ATP (CPM)	Age/gender of patients	Control (CPM)	ATP (CPM)
12 years, F	27.5 ± 4.7	202.8 ± 61.0	42 years, M	31.0 ± 2.1	1,648.5 ± 243.3
39 years, F	33.8 ± 15.3	173.5 ± 26.2	49 years, F	53.5 ± 32.2	882.0 ± 151.9
43 years, M	27.5 ± 5.1	170.0 ± 25.3	50 years, F	56.8 ± 22.8	1,537.0 ± 376.3
			65 years, F	27.3 ± 2.5	1,008.0 ± 198.6
			67 years, F	59.8 ± 35.2	874.3 ± 154.0

CMP, count per minute normalized against total protein levels; F, female; M, male.

### Comparison of osteoarthritis meniscal cell and osteoarthritis hyaline articular chondrocyte

**Figure 3:**
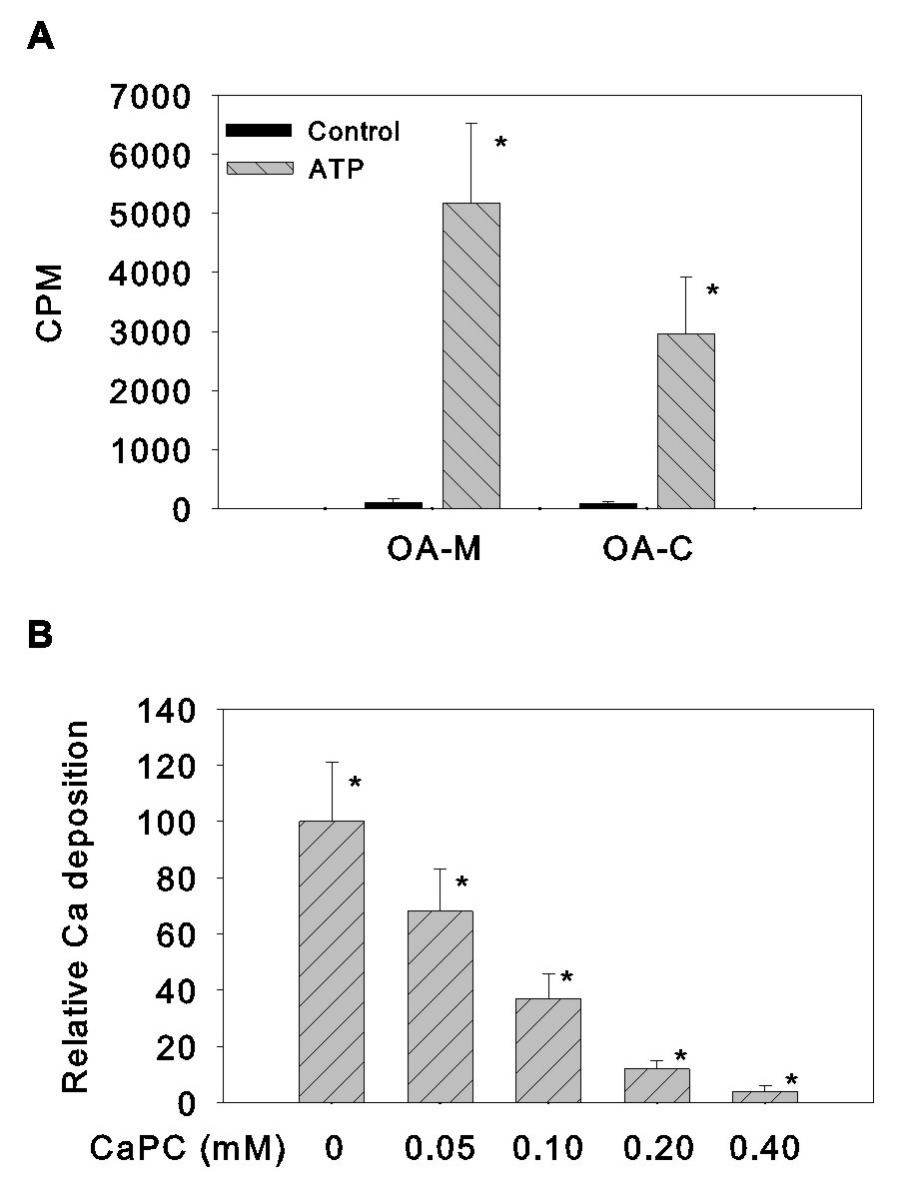
**Comparison of osteoarthritis meniscal cell-mediated and osteoarthritis chondrocyte-mediated calcium deposition**. **(a) **ATP-induced calcium deposition in the monolayer cultures of osteoarthritis (OA) meniscal cells derived from four OA patients (OA-M) (see Table 3) was 60% greater than present in the monolayer cultures of OA hyaline articular chondrocytes (OA-C). The difference was statistically significant (*P < 0.05). **(b) **Calcium phosphocitrate (CaPC) inhibited ATP-induced calcium deposition in the monolayer cultures of OA meniscal cells in a dose-dependent manner (*P < 0.001). Count per minute (CPM) data were normalized against total protein levels.

We compared cell-mediated calcium deposition between OA meniscal cells and OA hyaline articular chondrocytes derived from four OA patients. As shown in Figure 3a, both monolayer cultures of OA meniscal cells and OA hyaline articular chondrocytes produced large amounts of calcium deposition after the treatment with ATP for 72 hours. Collectively, OA meniscal cells produced more calcium deposition than OA hyaline articular chondrocytes. Finally, we found that CaPC, a potent anti-calcification agent, inhibited the OA meniscal cell-mediated calcium deposition in a dose-dependent manner (Figure 3b; *P *< 0.0001).

## Discussion

We demonstrated for the first time that calcium deposition was common in the menisci of end-stage OA patients. The ages of two OA patients were similar to the ages of two control subjects, and the ages of five among the eight OA patients were below the age of 60 years. Only OA meniscal specimens contained calcium deposits. This result indicates that meniscal calcification in OA is mainly a disease-related phenomenon. It is worth noting that no meniscal calcification is detected in nonselected cadavers before the age of 60 years [32].

Brandes and Muller examined meniscal chondrocalcinosis and found three types of meniscal calcification [44]. Type 1A was disseminated calcification, which affected all four menisci equally. Type 1B was calcification occurring in limited areas, which was associated with meniscal degeneration. Type 2 was a cloud-like diffuse calcification, which contained fine granular amorphous materials. The investigators concluded that type 1A calcification represented primary chondrocalcinosis, that type 1B calcification corresponded to secondary chondrocalcinosis, and that type 2 calcification was dystrophic and postnecrotic calcification.

In our study, we found three distinctive patterns of calcium deposition in the OA menisci. The first pattern of calcium deposition was calcification occurring in limited amounts, associated with meniscal degeneration (Figure 1g, h). This type of meniscal calcification is similar to the type 1B meniscal calcification described by Brandes and Muller [44]. The second pattern of calcium deposition was clusters of small-sized calcium deposits inside the meniscus (Figure 1i). This type of meniscal calcification appears to correspond to the type 2 meniscal calcification observed by Brandes and Muller [44]. The third pattern of calcium deposition was widespread clusters of medium-sized and large-sized calcium deposits. This type of calcification is probably a combination of the type 1B calcification and type 2 calcification in the more severe degenerative areas. Taken together, our findings suggest that meniscal calcification in OA may mainly correspond to dystrophic and secondary chondrocalcinosis rather than to primary chondrocalcinosis.

Calcium crystals were frequently found in the hyaline articular cartilage of end-stage OA patients [15-17]. It was believed that the hyaline articular cartilage was the most likely source of knee joint fluid crystals in OA patients. Degeneration of the hyaline articular cartilage would release the calcium crystals embedded in the cartilage into the knee joint fluid. In this study, we found that medium-sized and large-sized calcium deposits were commonly present at the degenerative edges (Figure 1g, h) or at the areas adjacent to the degenerative edges of OA menisci (Figure 1j). Because of their locations, these calcium deposits can be readily released into the knee joint fluid during joint articulation. Our findings suggest that degenerative menisci may be one of the sources of joint fluid crystals in OA.

Elevated gene expression of ANKH and ENPP1 causes crystal deposition in cartilage [25,45]. In the present study, we found that the expression of several genes implicated in the biomineralization biological process including ENPP1 and ANKH was upregulated in OA meniscal cells. This finding was consistent with the previous finding that ENPP1 was upregulated in the calcified regions of OA menisci [46] and that ANKH was upregulated in OA articular cartilage [47]. Our findings indicate that OA meniscal cells may play an active role in the pathological meniscal calcification. Indeed, OA meniscal cells induced much more calcium deposition than normal control meniscal cells in the monolayer cultures. This finding was consistent with a recent finding that OA hyaline articular chondrocytes produced calcium deposition in cell culture, whereas normal control hyaline articular chondrocytes derived from the hyaline articular cartilage of osteosarcoma patients did not [17]. The activities and protein levels of ENPP1, ANKH and matrix Gla protein in OA meniscal cells were not obtained in the present work. This information would certainly be interesting and important. We look forward to future study supplying this information.

The findings that calcium deposits were present in all OA menisci and that OA meniscal cells induced much more calcium deposition than normal meniscal cells will have significant impact on our understating of OA and the development of disease-modifying drugs for OA therapy. Recently, it was reported that CaPC, a potent anti-calcification agent, inhibited meniscal calcification in Hartley guinea pigs and that the inhibition was accompanied by a significant reduction in the degeneration of hyaline articular cartilage [48]. Our finding that CaPC inhibited OA meniscal cell-mediated calcium deposition was consistent with this report. Although our findings provide no support for the notion that calcium deposition in OA joint tissues is a causative factor to OA, pathological calcification in OA may still be a valid therapeutic target for OA therapy. Our study demonstrates clearly that meniscal calcification is a disease-related phenomenon in OA. Theoretically, inhibition of meniscal calcification can be achieved either by targeting the calcium deposits (physical target) or by targeting the cells (biological target). Targeting the calcium deposits directly will inhibit the growth of the calcium deposits and reduce the detrimental downstream biological effects of these calcium deposits. Targeting the cells at the cellular, genetic or epigenetic levels will not only inhibit the formation and growth of calcium deposits, but may also convert the altered OA meniscal cells to more normal-like meniscal cells, thereby eliminating an important disease component of OA.

Our study has some limitations that should be considered. The first limitation is that the normal control meniscal cells were not optimal normal meniscal cells. To minimize this limitation, we only collected overtly normal-appearing meniscal specimens from osteosarcoma patients whose tumors were located distant from the knee. Another limitation is the small size of our specimens; the exact contribution of aging to meniscal calcification could therefore not be determined. It is likely that an age-associated increase of meniscal calcification may account for some of the calcification in the clinical specimens. It is difficult to obtain age-matched control meniscal specimens because osteosarcoma occurs often in younger patients while OA occurs mostly in older patients. We will continue this line of study when more age-matched normal control meniscal specimens become available in the future.

## Conclusions

Our findings suggest that OA is not merely a hyaline articular cartilage disease, but also a meniscal disease. Pathological meniscal calcification mediated by OA meniscal cells, which may alter the biomechanical properties of the meniscus and the expression of extracellular matrix-degrading enzymes, is potentially an important contributory factor to OA.

## Abbreviations

ANKH: ankylosis, progressive homolog; ATP: adenosine-5'-triphosphate; CaPC: calcium phosphocitrate; DMEM: Dulbecco's modified Eagle's medium; ENPP1: ectonucleotide pyrophosphatase/phosphodiesterase 1; GAPDH: glyceraldehyde-3-phosphate dehydrogenase; OA: osteoarthritis; PCR: polymerase chain reaction; RT: reverse transcription.

## Competing interests

The authors declare that they have no competing interests.

## Authors' contributions

YS, HEG and ENH conceived the study and participated in its design and coordination. YS wrote the manuscript, and analyzed the microarrays. DRM and JSK provided surgical tissues and participated in the discussion of experimental results. HJN assisted with statistical analysis. NZ performed histologic embedding, sectioning and staining. YS and PRH graded alizarin red staining. PRH prepared cell cultures, performed the calcium deposition assay and extracted RNA. HEG assisted with manuscript preparation.
